# Erbium, Chromium-Doped Yttrium-Scandium-Gallium-Garnet Laser in Endodontics: A Narrative Review

**DOI:** 10.7759/cureus.108959

**Published:** 2026-05-16

**Authors:** Hazal Faiz Arslanparcasi, Mehmet Ali Arslanparcasi

**Affiliations:** 1 Endodontics, Harran University, Sanliurfa, TUR; 2 Dental Prosthesis Technology, Faculty of Vocational School of Health Services, Harran University, Sanliurfa, TUR

**Keywords:** endodontics, er:cr:ysgg laser, laser-activated irrigation, root canal disinfection, smear layer removal

## Abstract

Erbium, chromium-doped yttrium-scandium-gallium-garnet (Er,Cr:YSGG) laser-assisted endodontics has been investigated as an adjunctive approach to enhance irrigant activation, root canal disinfection, smear layer removal, and cleaning of complex anatomical areas. Owing to its wavelength of approximately 2780 nm, Er,Cr:YSGG laser energy is highly absorbed by water and hydroxyapatite, allowing interaction with both irrigation solutions and dental hard tissues. This interaction may generate photoacoustic streaming, cavitation-like fluid movement, microjet-like hydrodynamic effects, and localized photothermal effects within the root canal system.

This narrative review provides a descriptive and critical overview of the mechanisms, clinical applications, current evidence, and limitations of Er,Cr:YSGG laser-assisted endodontics. Available studies have evaluated its use in primary root canal treatment, retreatment procedures, regenerative endodontics, and endodontic microsurgery. Laboratory-based evidence suggests potential benefits in smear layer removal, debris reduction, antimicrobial activity, organic tissue dissolution, irrigant penetration into dentinal tubules, and obturation quality. However, most findings are derived from in vitro or ex vivo studies, and their direct translation to postoperative pain reduction, periapical healing, or long-term clinical success remains uncertain.

Current evidence indicates that the efficacy and safety of Er,Cr:YSGG laser-assisted protocols are strongly influenced by laser parameters, tip design, tip position, application time, irrigant type, canal anatomy, and operator experience. Therefore, Er,Cr:YSGG lasers should not be considered a replacement for conventional chemomechanical preparation, but rather as an adjunctive technology that may support standard endodontic protocols when used under appropriate safety-oriented conditions. Further well-designed clinical studies with standardized parameters and long-term outcomes are needed.

## Introduction and background

One of the main objectives of endodontic treatment is to remove microorganisms, necrotic tissue remnants, and biofilm structures from the root canal system as effectively as possible [1]. Mechanical preparation and chemical irrigation are essential components of this process; however, complete disinfection remains challenging because the root canal system contains isthmuses, lateral canals, apical ramifications, oval canals, dentinal tubules, and other anatomical irregularities that may not be adequately reached by conventional needle irrigation [2,3].

To overcome these limitations, several adjunctive irrigant activation methods have been investigated, including passive ultrasonic irrigation, sonic activation, negative-pressure irrigation, and laser-activated irrigation [4]. Among these approaches, laser-assisted endodontics has gained attention for its potential role in irrigant activation, biofilm disruption, and smear layer removal [5]. Nevertheless, laser applications should be considered supportive adjuncts to conventional chemomechanical preparation rather than independent treatment modalities [6].

Erbium, chromium-doped yttrium-scandium-gallium-garnet (Er,Cr:YSGG) laser-assisted protocols have been investigated in several endodontic applications, including primary root canal treatment, retreatment, regenerative endodontic procedures, and endodontic microsurgery [7,8]. However, current evidence remains heterogeneous in terms of laser parameters, energy settings, frequency, pulse duration, tip design, application time, irrigant type, and outcome measures. This variability limits direct comparison among studies and prevents definitive clinical recommendations [6].

Therefore, this narrative review provides a descriptive and critical overview of the mechanisms, clinical application areas, current evidence, and limitations of Er,Cr:YSGG laser-assisted endodontics. Particular emphasis is placed on distinguishing laboratory-based findings from clinically validated outcomes and on evaluating Er,Cr:YSGG lasers as adjunctive tools rather than replacements for standard chemomechanical preparation.

## Review

Methodological approach of the narrative review

This study was designed as a narrative review to evaluate the clinical applications, current evidence, and limitations of Er,Cr:YSGG laser-assisted endodontics. The review was not designed as a systematic review or meta-analysis. Therefore, no quantitative synthesis, meta-analysis, meta-regression, formal risk-of-bias assessment, or evidence grading was performed. Instead, the available literature was narratively synthesized to provide a descriptive and critical overview of the topic. Because this article was designed as a narrative review rather than a systematic review, the study selection process was not presented using a Preferred Reporting Items for Systematic Reviews and Meta-Analyses (PRISMA) flow diagram. However, the search strategy, eligibility approach, and narrative synthesis process were clarified to improve methodological transparency.

A structured literature search was conducted in PubMed, Web of Science, Scopus, and Google Scholar, with the final literature check completed in May 2026 before manuscript submission. The search was limited to English-language publications, and no restriction was applied regarding study design. The publication period included studies published up to May 2026. The search strategy focused on studies evaluating Er,Cr:YSGG laser-assisted protocols in endodontic applications. The following search terms were used either individually or in Boolean combinations: “Er,Cr:YSGG,” “erbium chromium yttrium scandium gallium garnet,” “2780 nm laser,” “endodontics,” “laser-assisted endodontics,” “laser-activated irrigation,” “root canal disinfection,” “Enterococcus faecalis,” “biofilm,” “smear layer,” “dentin microhardness,” “bond strength,” “postoperative pain,” “retreatment,” “regenerative endodontics,” and “endodontic surgery.”

The main Boolean search strategy was as follows: (“Er,Cr:YSGG” OR “erbium chromium yttrium scandium gallium garnet” OR “2780 nm laser”) AND (“endodontics” OR “root canal” OR “root canal disinfection” OR “laser-activated irrigation” OR “smear layer” OR “biofilm” OR “Enterococcus faecalis” OR “retreatment” OR “regenerative endodontics” OR “endodontic surgery”).

Peer-reviewed in vitro studies, ex vivo studies, clinical studies, randomized clinical trials, systematic reviews, and relevant narrative reviews were considered eligible if they evaluated the role of Er,Cr:YSGG lasers in endodontic applications. Studies were included when they addressed root canal disinfection, irrigant activation, antimicrobial efficacy, biofilm disruption, smear layer removal, dentin surface effects, obturation-related outcomes, retreatment procedures, regenerative endodontics, postoperative outcomes, or endodontic surgery.

Studies were excluded if they were unrelated to endodontics, focused exclusively on other laser systems without direct relevance to Er,Cr:YSGG-assisted endodontics, lacked sufficient methodological or outcome information, or were only marginally related to the clinical scope of this review. Comparative studies involving Er,Cr:YSGG lasers and other irrigation activation methods were considered when they contributed to the interpretation of the current evidence.

The literature was screened at the title, abstract, and full-text levels according to relevance to the review aim. The included studies were then narratively synthesized under the following themes: mechanisms of action, root canal disinfection, antimicrobial efficacy, biofilm disruption, laser-activated irrigation, smear layer removal, dentin surface and mechanical effects, clinical applications, postoperative outcomes, safety considerations, and methodological limitations. Greater interpretive emphasis was placed on clinical studies, systematic reviews, and studies directly evaluating Er,Cr:YSGG-assisted endodontic protocols. Laboratory-based findings were interpreted cautiously and were not considered equivalent to clinical outcomes such as postoperative pain reduction, periapical healing, tooth survival, or long-term treatment success.

Principles of Er,Cr:YSGG laser technology in endodontics

Er,Cr:YSGG lasers represent an erbium laser system that differs from low-level laser therapies primarily used for photobiomodulation, analgesia, and wound healing. In endodontics, Er,Cr:YSGG lasers are mainly evaluated for their interaction with irrigation solutions and dental hard tissues rather than for biostimulatory effects [9,10]. Therefore, their relevance in root canal treatment is principally related to irrigant activation, smear layer management, dentin surface modification, and disinfection support.

Wavelength and Tissue Interaction

Er,Cr:YSGG lasers operate at a wavelength of approximately 2780 nm, which is highly absorbed by water and hydroxyapatite [5]. This absorption profile enables the laser energy to interact with both irrigation solutions and root canal dentin. In the presence of irrigants, this interaction may enhance fluid movement and support cleaning of areas that are difficult to reach with conventional irrigation. In contact with dentin, however, the same interaction may also produce surface alterations depending on the selected parameters [6].

The clinical effect of Er,Cr:YSGG laser application is influenced by energy level, pulse duration, application time, tip position, irrigant presence, and tissue moisture [4]. Inappropriate parameter selection may lead to undesirable dentin changes, including increased roughness, carbonization-like alterations, microcrack formation, or residual debris, particularly in the apical region [11]. Therefore, parameter optimization is essential to balance cleaning efficacy with the preservation of dentin integrity [12].

Photoacoustic and Photothermal Effects

The main mechanisms of Er,Cr:YSGG laser-assisted irrigation are associated with photoacoustic and photothermal effects. Absorption of laser energy within the irrigant may cause rapid vapor bubble formation, followed by expansion and collapse. This process can generate cavitation-like fluid movement, shock waves, and microjet-like hydrodynamic effects within the root canal space [1]. These fluid dynamics may increase irrigant penetration into anatomical irregularities and support smear layer removal and biofilm disruption, particularly in areas that are difficult to access mechanically [13].

The photothermal effect may contribute to antimicrobial activity through localized temperature elevation. However, excessive heat generation may compromise periodontal tissues and dentin integrity. Therefore, temperature control, water cooling, appropriate energy selection, and limited application times are important safety considerations during Er,Cr:YSGG laser-assisted protocols [14,15].

Parameters Affecting Clinical Performance

The clinical performance and safety of Er,Cr:YSGG laser-assisted endodontic applications depend on multiple parameters, including energy level, power setting, pulse duration, frequency, tip design, tip position, distance from the working length, application time, irrigant type and concentration, canal anatomy, apical diameter, tissue moisture, and operator experience [13]. These variables may influence antimicrobial efficacy, irrigant activation, smear layer removal, dentin surface changes, apical extrusion risk, and thermal safety. For example, energy level and tip design may affect irrigant movement and canal wall cleaning, whereas certain tip designs or deeper insertion levels may increase apically directed fluid movement [16].

Pulse frequency and pulse duration are particularly relevant to the formation of photoacoustic streaming. Short pulse durations and appropriate frequency settings may enhance fluid dynamics, whereas prolonged application time or excessive energy density may increase thermal risk [4,14]. Similarly, tip position should be standardized because placement close to the working length may influence apical cleaning but may also increase extrusion-related safety concerns, especially in teeth with immature apices, resorption, or wide apical foramina [16].

Canal anatomy is another determinant of clinical performance. Narrow, curved, or anatomically complex canals may limit tip placement and reduce the uniform distribution of irrigant activation. Operator experience is also important for selecting appropriate settings, controlling tip movement, and maintaining a safety-oriented protocol [12].

Overall, Er,Cr:YSGG laser-assisted protocols require careful standardization of laser parameters, irrigation conditions, and clinical application techniques. Current heterogeneity in study designs and outcome measures limits direct comparisons and prevents definitive clinical recommendations [12,13].

Clinical applications of Er,Cr:YSGG laser-assisted endodontics

Er,Cr:YSGG laser-assisted endodontic protocols have been investigated as adjunctive approaches in various clinical application areas, including primary root canal treatment, retreatment procedures, regenerative endodontics, and endodontic microsurgery [5].

In primary root canal treatment, Er,Cr:YSGG laser-assisted irrigation has primarily been evaluated during the final irrigation activation phase. Reported effects include support for root canal disinfection, smear layer removal, debris reduction, irrigant penetration into dentinal tubules, and cleaning of complex anatomical areas [17]. These effects are mainly associated with vapor bubble formation, photoacoustic streaming, cavitation-like fluid movement, and microjet-like hydrodynamic effects generated by the absorption of laser energy within the irrigation solution [4].

Smear layer removal is one of the most frequently investigated outcomes of Er,Cr:YSGG laser-activated irrigation. The smear layer may contain organic and inorganic debris, dentin particles, pulp remnants, and microorganisms, and may limit irrigant penetration, reduce disinfection efficacy, and interfere with sealer adaptation. Er,Cr:YSGG laser-assisted protocols have generally been evaluated in combination with 17% ethylenediaminetetraacetic acid (EDTA), 2.5-5% sodium hypochlorite (NaOCl), or combined irrigation regimens. Some studies have reported that Er,Cr:YSGG laser activation with EDTA may produce cleaner dentin surfaces than EDTA alone [18].

However, smear layer removal varies across canal regions. More consistent results may be observed in the coronal and middle thirds, whereas efficacy in the apical third may be more variable because of canal anatomy, curvature, narrow canal diameter, and limited irrigant access [17]. Power setting, application time, and irrigation protocol may also affect the outcome. Therefore, smear layer removal should be interpreted as a laboratory indicator of canal cleanliness and not as direct evidence of clinical success, periodontal healing, or long-term prognosis [1].

Er,Cr:YSGG laser activation may also enhance irrigant and sealer penetration into dentinal tubules. Dentinal tubules may serve as reservoirs for microorganisms, biofilm remnants, and organic debris after chemomechanical preparation. Improved penetration may therefore support deeper disinfection and filling material adaptation [19]. Confocal microscopy studies have reported that final irrigation protocols using Er,Cr:YSGG laser activation may improve sealer penetration into dentinal tubules [20]. Nevertheless, these findings remain surrogate laboratory outcomes and require cautious clinical interpretation.

In terms of antimicrobial efficacy, Arnabat et al. evaluated Er,Cr:YSGG laser activity in human root canals experimentally colonized with *Enterococcus faecalis*. Although 5% NaOCl was the most effective application, Er,Cr:YSGG laser irradiation at 2 W for 60 seconds showed bactericidal efficacy comparable to that of 5% NaOCl [21]. This finding supports the potential adjunctive antimicrobial role of Er,Cr:YSGG lasers, but does not indicate that laser application can replace NaOCl-based irrigation.

Regarding organic tissue dissolution, Liu et al. reported that two Er,Cr:YSGG laser systems used with 3% NaOCl provided faster tissue dissolution than conventional needle irrigation and passive ultrasonic irrigation. However, the multisonic ultracleaning system showed a higher tissue dissolution rate than the laser systems [22]. Therefore, Er,Cr:YSGG activation may be effective under selected experimental conditions, but current evidence does not support its universal superiority over other irrigation activation methods.

The effect of Er,Cr:YSGG laser-activated irrigation on obturation quality has also been investigated. Yamaguchi et al. compared Er,Cr:YSGG laser activation with sonic activation in conservatively prepared mandibular molars with isthmuses using micro-CT. The percentage of unfilled voids was 17.24% in the sonic group and 10.73% in the laser group, with a statistically significant difference [23]. This finding suggests that Er,Cr:YSGG laser activation may support obturation quality by improving cleaning in unprepared anatomical areas; however, because the study was ex vivo, these results should not be directly translated into long-term clinical success.

In root canal retreatment, residual filling materials, sealer remnants, debris, persistent biofilm residues, and difficult-to-reach anatomical areas remain important clinical challenges [24]. Er,Cr:YSGG laser-assisted protocols have been evaluated as adjunctive methods to improve cleaning after rotary retreatment systems. Some studies have reported cleaner surface findings in the coronal and middle thirds, whereas efficacy may be more limited in the apical region [7]. Nevertheless, no available protocol has demonstrated complete cleaning. Thermal effects associated with high power settings and undesirable dentin surface alterations have also been reported [25]. Therefore, Er,Cr:YSGG lasers may support rotary systems and irrigation protocols in retreatment, but should not be considered independent retreatment methods.

In regenerative endodontic procedures, immature teeth, open apices, the apical papilla, stem cell viability, and periapical tissue safety are major considerations. Therefore, Er,Cr:YSGG laser-assisted applications should be evaluated with particular caution in this context [26]. Ex vivo models have highlighted the risk of irrigant extrusion. Vapor bubble dynamics generated during laser-activated irrigation may increase apically directed fluid movement and may create biological safety concerns if toxic irrigants are transported into the periapical tissues [27]. Because clinical data remain limited, further studies are needed to evaluate low-energy protocols, stem cell viability, root dentin biocompatibility, and periapical tissue safety.

In endodontic microsurgery, Er,Cr:YSGG lasers have been investigated for root-end surface management, smear layer reduction, hard-tissue ablation, and retrograde cavity preparation. Shetty et al. compared root-end cavity preparation using Er,Cr:YSGG laser, ultrasonic retrotip, and bur, and evaluated apical microleakage after retrograde filling. Their findings suggest that Er,Cr:YSGG laser may be considered as one possible method for retrograde cavity preparation [28]. However, evidence in this area remains largely based on limited experimental models, and randomized clinical trials evaluating Er,Cr:YSGG lasers in endodontic microsurgery are still insufficient. Therefore, potential technical advantages should be interpreted cautiously until stronger clinical evidence becomes available.

Apical safety is a key issue in Er,Cr:YSGG laser-activated irrigation. Although laser-induced fluid movement may improve irrigant activation and canal wall cleaning, it may also increase the risk of apical irrigant extrusion, debris extrusion, and periapical tissue irritation. Extrusion of biologically irritating solutions such as NaOCl into the periradicular tissues may be associated with postoperative pain, inflammatory response, and delayed healing [29]. This risk is particularly relevant in open-apex teeth, immature roots, apical resorption, wide apical diameters, and teeth with periapical lesions. In such situations, tip position, distance from the working length, energy level, pulse frequency, application time, irrigant volume, and apical canal diameter should be carefully controlled [30].

Overall, current evidence suggests that Er,Cr:YSGG laser-assisted protocols may provide promising laboratory-based effects, particularly in smear layer removal, irrigant activation, antimicrobial activity, tissue dissolution, dentinal tubule penetration, and obturation-related outcomes. However, many of these findings are derived from in vitro or ex vivo models and should not be considered equivalent to clinical outcomes such as reduced postoperative pain, improved periapical healing, or long-term treatment success.

## Conclusions

Er,Cr:YSGG laser-assisted endodontics may serve as a supportive adjunct to conventional chemomechanical preparation by enhancing irrigant activation, smear layer removal, debris reduction, antimicrobial activity, tissue dissolution, dentinal tubule penetration, and obturation-related outcomes under selected experimental conditions. However, the current evidence is largely based on in vitro and ex vivo studies, and these findings should not be directly interpreted as evidence of reduced postoperative pain, improved periapical healing, or long-term clinical success.

The clinical performance and safety of Er,Cr:YSGG laser-assisted protocols depend on several variables, including energy level, pulse frequency, tip design, tip position, distance from the working length, application time, irrigant type, canal anatomy, apical morphology, and operator experience. Therefore, Er,Cr:YSGG lasers should not be considered a replacement for standard irrigation or mechanical preparation, but rather as an adjunctive technology requiring careful parameter selection and safety-oriented clinical application.

Future research should prioritize well-designed clinical studies with standardized laser parameters, clearly defined irrigation protocols, clinically meaningful outcome measures, and long-term follow-up data.
